# Etiopathogenesis of Suicide: A Conceptual Analysis of Risk and Prevention Within a Comprehensive, Deterministic Model

**DOI:** 10.3389/fpsyg.2019.02087

**Published:** 2019-09-12

**Authors:** Jack C. Lennon

**Affiliations:** ^1^Department of Psychology, Adler University, Chicago, IL, United States; ^2^Section of Parkinson’s Disease and Movement Disorders, Department of Neurological Sciences, Rush University Medical Center, Chicago, IL, United States; ^3^Department of Behavioral Sciences, Rush Neurobehavioral Center, Rush University Medical Center, Skokie, IL, United States

**Keywords:** suicide, neurosciences, preventive psychiatry, psychological theory, nomenclature, etiopathogenesis

## Abstract

Suicide is a rising global health concern receiving disproportionate attention in comparison to other health conditions. In spite of substantial technological and scientific advancements, suicide research has continued to move slowly in terms of clinical translation due to the complexity of neural mechanisms, and subjective experiences that seem to underpin this complex human behavior. This paper analyzes the concepts of risk and prevention in the context of suicide in an attempt to bridge the large methodological and theoretical gaps between the biological, psychological, and sociological dimensions. This paper aims to accomplish the following objectives: (1) operationalize the concepts of suicide risk and prevention as they relate to current knowledge and capabilities; (2) synthesize and integrate suicide research across biological, psychological, and sociological dimensions; (3) discuss limitations of each dimension in isolation; (4) suggest a model of etiopathogenesis that incorporates extant literature and bridges unnecessary gaps between dimensions; and (5) suggest future directions for multidimensional research through the inclusion of principles from the physical sciences. Ultimately, this paper provides a basis for a comprehensive model of suicide within a deterministic, chaotic system.

## Introduction

Human life does not occur within a vacuum, such that metabolic, hormonal, and all biological processes are impacted by factors external to individual organ systems and the body as a whole. The external, socioeconomic and political environments in which the individual resides significantly interact with these biological processes and, in turn, biological processes can manifest in behaviors that either avoid or perpetuate these external factors. There are countless but finite factors involved in human life that result in a finite set of outcomes. For these reasons, the study of human behavior is the domain harboring some of the most complex questions of life. Empirical findings are difficult to translate into clinical practice because of the broad-range, synergistic activity across biological, psychological, and sociological dimensions. This exceedingly disconcerting formula has resulted in scientific efforts to answer chicken-and-egg questions that may be less important to the overall permutation than the realization that events are recursive and bidirectional. It is interesting to question what could be accomplished if a complex human behavior such as suicide was discussed in terms of its etiopathogenesis rather than multiple non-integrative models that de-medicalize a serious global health concern.

Suicide is a human behavior that is arguably one of the most difficult to confront clinically, in spite of ample literature on the topic. As the tenth-leading cause of death in the United States, observed suicide rates are currently higher than they have been since World War II (Drapeau and McIntosh, 2017; Centers for Disease Control and Prevention, 2018). Suicide research spans an array of perspectives, all contributing useful findings to the field but, ultimately, has not resulted in reducing suicide deaths. While suicide is generally viewed as a preventable cause of death, there is little evidence to suggest that our translational abilities are sufficient for prevention. To methodically prevent a fatal behavior, it is necessary to understand one’s risk of engaging in the behavior. A global effort to modify or reduce risk factors, such as a program of theoretical prevention, would underestimate the role of biology; focusing only on biological components of suicide, conversely, would neglect socially-induced biological and epigenetic alterations. Established risk factors have been discovered across all domains of study but have also proven insufficient or unnecessarily sensitive to non-life-threatening ideation. Given the difficulty incorporating disciplines of research within a clinically-oriented biopsychosocial model of human life, suicide cannot be reported as a preventable cause of death in its current state. Instead, it is an individual-dependent scientific quandary currently beyond the reach of primary and secondary prevention efforts. This state of affairs, however, is neither a point of stagnation nor a condition of insufficient findings – it is a result of a failure to promote adequate study designs that establish common ground among theoretical and scientific viewpoints (Sica, 2017; Roberts et al., 2019), all of which possess reconcilable differences and, in fact, positively contribute to one another.

There exist significantly large theoretical, methodological, and phenomenological gaps between the biological, psychological, and sociological sciences in the domain of suicide research. While it is well-recognized that neurologic and psychiatric conditions are prominent in present-day, many scholars continue to harbor beliefs about a mind-brain dichotomy that essentially absolves science of the burden of seeking viable models of human behavior in the present moment (Silbersweig, 2005). This dichotomy fallaciously subsumes the beliefs that an individual can process information without the brain, can act autonomously, and is conscious of his or her true risk of suicide, in spite of evidence that those who die by suicide are neurobiologically distinct from those who experience depression and suicide ideation (SI) but do not die by suicide (Pitchot et al., 2001; Colle et al., 2015). The ongoing debate regarding personal agency and conscious behavior is heavily implicated in this discussion, leading to significant disagreements and biases that may perpetuate variability in methodological approaches to this global health concern.

If the complexity of a problem is negatively correlated to the interdisciplinary efforts utilized to discover its solutions, suicide deaths may very well continue to rise. Regardless of perspective, reduction to one perspective implies that life is, in some fashion, occurring within a vacuum. Even psychological and sociological endeavors are highly limited in spite of their efforts to incorporate external factors, as they frequently disregard the bidirectional relationship between their respective variables and human biology (Gould et al., 2017). In psychology and sociology, the environment serves as the meta-vacuum with biological sequelae serving as only byproducts without any executive control of or impact on its environment. This, in itself, is reductionism disguised as an overarching, all-encompassing theory that accounts for all variables impacting human life. However, this is not a biopsychosocial model that serves to incorporate all necessary scientific findings – it is one that believes psychosocial components impact biology without reciprocal damage. Each perspective serves as a necessary but insufficient component of a suicide model.

This conceptual analysis aims to accomplish the following objectives: (1) operationalize the concepts of suicide risk and prevention as they relate to current knowledge and capabilities; (2) synthesize and integrate suicide research across biological, psychological, and sociological dimensions; (3) discuss limitations of each dimension in isolation; (4) suggest a model of etiopathogenesis that incorporates extant literature and bridges unnecessary gaps between dimensions; and (5) suggest future directions for multidimensional research. Appealing to the evidence, it is argued that only through a deep analysis of current concepts in the context of suicide research that we may gain insight into that which has yet to be discovered – insight that could result in a comprehensive, deterministic model of suicide and, subsequently, quantitatively viable primary and secondary prevention efforts.

## Conceptualizing Suicide Risk and Prevention

Philosophical and religious positions on suicide, which will henceforth be defined as the act of ending one’s own life through seemingly intentional means, have undergone only minor revisions since the times of early philosophers, and religious doctrines. Camus (2005) noted that the questions of suicide and the meaning of life may be viewed as primary, while ethics, knowledge, and truth remain secondary to these ultimate questions. This suggests that suicide has been a philosophical priority across history, but a major concern is its placement alongside such a complex and subjective topic as the *meaning of life.* Suicide is irrefutably complex and poses a formidable barrier to clinical translation but, with this implied level of philosophical complexity that has inexplicably survived over time, empirical inquiry can become misguided. Falsifiable philosophical questions guide science and, without accurate and testable conceptualizations of suicide, research methodology is at risk of falling short.

Neuroscientific findings, which will be synthesized in the subsequent section, have elucidated the role of the brain in behavioral, cognitive, and affective processes. As the organ responsible for behavior and the residence of cognitive and emotional states, it is also the source of suicidal thoughts and behaviors (STB), SI, and the capacity for suicide attempts. While human behavior may appear to result from personal autonomy, behaviors are sequelae of complicated sequences of events that impact individuals differently. Suicide, in particular, is often a behavioral byproduct of negative affective states that is deemed preventable by those who simultaneously view it as impervious to quantitative prediction. Whether we are discussing suicide or cigarette smoking, that which is preventable logically depends on our ability to understand it as not only a discrete behavior or event, but a result of many events that may, itself, be a process both created and maintained until enacted to its fullest extent. For suicide to be preventable solely within the affected individual, he or she must be a free, conscious agent of behavior. Even if this were to be true, however, negative emotional states stemming from subcortical regions can transform irrational or stigmatized thoughts and behaviors into seemingly rational solutions to ongoing stressors. To state that one has equal opportunity to choose any option deemed plausible by outside observers demonstrably undermines unique differences in individual lives and the brains that adapt to these circumstances. When brain regions are unavailable or inoperable, the processes for which they are necessary fail to be engaged. It is certainly true that the brain can adapt to disruption due to processes such as procedural memory being diffuse rather than localized, but decision-making and overall functioning are invariably altered.

Knowing that an affected individual cannot take on the role of prevention himself or herself, the proponents of current prevention methods must be recruiting outside observers for this role. As a relevant thought experiment, we can imagine a depressed individual on the edge of a cliff with rational observers in the area. This individual could theoretically be pulled from the edge by an onlooker and may report a negative emotional state, but whether or not he or she would have taken the fall is an unfalsifiable question. There are substantial differences between active suicide ideators and attempters, which are analogous to the inexplicable differences between cigarette smokers who do and do not develop lung cancer. A history of attempts is a risk factor for future attempts, but not everyone engages in numerous attempts and majority of suicide deaths are first-attempters, particularly when utilizing a firearm (Bostwick et al., 2016). In a recent study, for example, only age and subsequent treatment significantly distinguished between those who attempted once and those who attempted on multiple occasions (Defayette et al., 2019). While this suggests tremendous uncertainty when viewing a single individual’s risk, it also suggests that treatment can serve as a positive perturbation in the life trajectory and, in a sense, the pathogenesis of suicide.

Suicide being viewed as a preventable cause of death implies quite directly that either the suicidal individual or those close to the suicidal individual are able to determine risk. This may be in the form of referring a depressed family member to the appropriate services, thereby potentially preventing a future suicide attempt. However, this is not the same concept as suicide prevention – awaiting noticeably symptomatic depression would likely lead to a marginal change in suicide rates. These impromptu thought experiments suggest that current views of prevention are circumstantial and unfalsifiably preventative because we cannot currently determine with certainty based on visual signs the individuals who have the unique set of factors that would result in a true suicide attempt. As an extreme example, it would be unnecessary and potentially severely consequential to hospitalize all individuals presenting with depression. While increases in treatment accessibility and compliance are certainly positive promoters of change, they serve as necessary but insufficient bandages that do not directly confront the issue of suicide.

## Dimensions of Suicide

### Psychological Findings

Psychological science is a major force in suicide research due to the clear affective states that contribute to risk and the therapeutics known to reduce this risk. The study of the mind, or psyche, must not be misinterpreted within the context of current knowledge and should be, instead, understood as an approach to understanding subjective human well-being and suffering. This subjectivity and ambiguity exist in a relationship with all other components that make us uniquely human and are only created through the processing of stimuli – without a capacity to process information, subjective meaning cannot be derived. All components of a life trajectory such as age (Drapeau and McIntosh, 2017; Conejero et al., 2018b), ethnicity (Curtin et al., 2016), socioeconomic status (Lewis et al., 1988; Lee et al., 2017), gender (Freeman et al., 2017) and transgender (Testa et al., 2017), disability (Lund et al., 2015), health concerns (Ahmedani et al., 2017), trauma history (de Mattos Souza et al., 2016), marital status (Kposowa, 2000), stigma and social acceptance (Schomerus et al., 2014), access to healthcare (Tondo et al., 2006), access to toxic substances such as pesticides (Kong and Zhang, 2010), and countless others contribute to suicide risk. Each of these must have an effect on the individual if it is to impact suicide risk.

Regarding suicide risk assessment, self-report measures of risk and clinician judgment have been deemed insufficient and statistically comparable to chance (Nock et al., 2012), with clinicians often falling prey to biases when evaluating patients, such as when there is a difference in age (Berman et al., 2016). In fact, Nock et al. (2012) found that judgment and evaluation of risk factors are more predictive of SI than risk of suicide attempts. Further, Barlow (2016) reported that suicide assessment knowledge among relevant clinicians is neither universal nor necessarily adequate and is further compromised by the time permitted to assess for risk. Significant efforts are being made to stray from self-report measures, as they are based on the false premise that an individual can accurately and honestly report his or her current affective and cognitive states. In this process, researchers have pursued implicit association tests (IATs) that gauge current affective and cognitive processes occurring in the brain that are beyond conscious awareness with much success (Nock et al., 2010).

Glenn et al. (2017) found that implicit associations related to self-harm were stronger in those with histories of suicide attempts as well as those with more severe non-suicidal self-injury (NSSI). While various IATs have been utilized to gauge suicidal behaviors, the death/suicide IAT (d/sIAT) has proven to be a significant predictor of suicide risk compared to current measures (Barnes et al., 2017). In individuals presenting with suicidal ideation, the d/sIAT combined with a history of NSSI has been associated with a 600% increase in odds of attempting suicide prior to 6-month follow-up (Nock et al., 2010), suggesting that even a combination of risk factors can be confronted to predict true suicide risk that still appeal to behavioral, cognitive, and affective processes occurring within the brain. For example, it has been found that those with a history of suicide attempts appeared to be driven more by the specific intent to engage in the suicidal act than social isolation, arousal, or rumination related to other factors (Rogers and Joiner, 2018a, b). However, this measure may also fall short regarding other STBs in spite of its efforts, as results from the d/sIAT are not always associated with lifetime depression or SI (Chiurliza et al., 2018).

While IATs seem to show promise in distinguishing between ideators and future attempters, one must question which brain regions, maps, and neural circuits are implicated in these suicide-affirmative item responses. These findings may provide reassurance to those who believe that negative emotional states are “felt” by a distinct entity with the capacity to communicate with the brain and body, however, this does not add to the argument for dichotomy. Instead, IATs serve as a stepping stone in deviating from unreliable self-reports and providing evidence for brain activity that may still be beyond our technological abilities to quantify or detect with expediency. Even if the mind were to exist outside of the brain, its own activity would be contingent to some degree on neural mechanisms that could reasonably inform of suicide risk just as well as observable, negative emotional states.

These statements are corroborated by dualistic theories of implicit affective processes, such as those studied and disseminated by Schore (2002, 2009a,b, 2011) in which neural mechanisms underlie subjective experiences. Emotional states certainly play a meaningful role in human behavior, whether it is related to personal taste preferences (Lee et al., 2006), sexual attraction (Dutton and Aron, 1974), risk-taking (Heilman et al., 2010; Ackerman et al., 2015), or the use of figurative language (Fussell and Moss, 1998). Research also suggests that social support is a protective factor, but it does not prove sufficient for prevention because its role is mediated by other factors (Kleiman and Riskind, 2013; Zhang and Lin, 2015). Protocols have been established that attempt to expedite and standardize this process of risk detection, such as that posed by Joiner et al. (1999). The common data elements (CDEs), which assess risk factors, is another valid method currently being utilized to routinize the process of assessment (Ringer et al., 2018). This is a necessary paradigm shift that harbors the potential to drastically alter psychiatry, psychology, and other fields examining brain function (Silbersweig, 2005).

Joiner (2005) has developed the interpersonal-psychological theory of suicide, which has led to remarkable promise related to recognizing those at risk for suicide. In his final pathway model, it is suggested that two psychological components are necessary for an individual to attempt suicide: (1) thwarted belongingness and (2) perceived burdensomeness (Joiner et al., 2002, 2009). Along with these components, and arguably one of the most insightful and useful views on suicide to-date, an individual must harbor a state of fearlessness that permits such a serious and fatal behavior. In this manner, the theory abolishes the idea of impulsivity and non-premeditated action and, instead, proposes the unrefuted premise that a suicide attempter is highly focused, attentive, and organized. The intensity of focus required has been found evident in significantly reduced blink-rate in individuals immediately before their deaths (Joiner et al., 2016). The exact time and nature of the behavior may not align perfectly with one’s plans and are likely dependent on his or her trajectory, but the intent is present to such a degree that a hesitant or scared individual would likely be incapable of following through with the act.

#### Impulsivity in the Context of Suicide

*Impulsivity* is a concept without a universal definition and is utilized across scientific disciplines in much the same manner as other seemingly self-evident terms. In the domain of suicide research, this misunderstanding poses a major obstacle in both the scientific community as well as the general public. In Neuropsychology, for example, impulsivity suggests bottom-up processing that is in reaction to either external or internal stimuli (i.e., danger) and is a component of executive dysfunction that suggests a tendency to act without premeditation due to issues with disinhibition (Lezak et al., 2012). The basic definition of impulsivity, then, can easily lead one to believe that suicide is unpredictable and a consistent risk in individuals who are depressed, possess a history of attempts, or engage in NSSI. While these are certainly risk factors, it has been well-established that those who die by suicide engage in premeditation for weeks, months, and even years prior to engaging in an attempt (Anestis et al., 2014). In fact, impulsive attempts have been found to be less lethal and inversely related to intent to die (May and Klonsky, 2016).

### Sociological Findings

Durkheim (1897) was an early pioneer in suicide research who was opposed to the theory of imitation, observing that deaths decreased during times of tragedy due to a perceived avoidance of isolation or detachment from society as a whole (Abrutyn and Mueller, 2014b). Over time, a wide range of studies focusing on sociological variables have noted increased risk based on an array of external factors. For example, social relationships have been reported as significant risk factors for suicide attempts when those interactions involve the discussion of suicidality, increasing vulnerability in adolescents (Abrutyn and Mueller, 2014a). Further, bullying related to sexual orientation and ethnicity is a risk factor for suicide located outside the self – in the hands of others – but results in increased risk of SI and issues related to identity (Mueller et al., 2014). The content of social interaction, then, is more important from a predictive standpoint than the presence of social interaction and belongingness.

Researchers including Wray et al. (2011) have also noted the flaws in micro-macro research, stating the importance of multidisciplinary efforts in the context of suicide research. Contagion has been a pronounced topic in recent years with ongoing media coverage related to suicidality. This spread of a seemingly viable coping strategy for distress is particularly problematic among the most cognitively vulnerable populations and can be spread through social media outlets and exposure to topics of suicide have been found to be associated with an increased risk of subsequent attempts (Yildiz et al., 2019). This concept of acquiring an idea that seems to assist with resolving dissonance and distress becomes more recognizable through research on Strain Theory, such that psychological strain is reported in suicide notes with the most common complaint being coping difficulties (Zhang et al., 2018).

Results have been mixed regarding whether or not some of the claims related to contagion can be extended to fictional media, in spite of numerous claims that television and movies result in a widespread increase in suicide risk for children and adolescents (VanderWeele et al., 2019). However, the *diffusion* of suicide through social means following exposure is certainly an observable phenomenon that is an important consideration for media and the general public alike, particularly as it relates to community prevention efforts (Abrutyn et al., 2019).

### Neurobiological Findings

Neuropsychological tests and neuroimaging techniques are not standards of care for suicidal individuals presenting to medical settings (Homaifar et al., 2012a). These evaluative tools do not possess reliable predictive capabilities as stand-alone measures in emergency settings and require extensive time to execute. In spite of an apparent lack of clinical utility, particularly during acute crises, neurobiology is a critical component of the life trajectory, and can provide insight into how the brain processes both external and internal stimuli.

The understanding of underlying organic disturbances in the brains of suicidal individuals are relatively ubiquitous in suicide research. Suicide is generally a partial result of negative emotional states that impact an individual at molecular, functional, structural, and behavioral levels. Correlates of suicide risk have been discovered implicating executive functioning (Burton et al., 2011; Keilp et al., 2013; Gujral et al., 2014), sympathetic nervous system dysregulation (McGirr et al., 2010), cognitive inflexibility (Miranda et al., 2012), and specific decision-making abilities (Hoehne et al., 2015; Deisenhammer et al., 2018). Neurobiological correlates were found in studies revealing that glutamatergic and GABAergic genes undergo significant alterations in depressed individuals as well as those who die by suicide compared to healthy controls (Sequeira et al., 2009; Zhurov et al., 2012; Zhao et al., 2018).

In the field of epigenetics, Haghighi et al. (2014) found an increase in DNA methylation in brains of suicidal individuals, which suggests that one’s genes are fluid and are impacted over the course of life beyond conscious awareness. Neural circuitry has been found dysfunctional in individuals exhibiting suicidal behavior through neuroimaging of humans (Soulas et al., 2008; Zhang et al., 2014) and dysregulation of the infralimbic cortex in rats has led to conclusions related to unconscious impulsivity and disinhibition (Tsutsui-Kimura et al., 2016). Postmortem samples of three specific genes associated with the hypothalamic-pituitary-adrenal (HPA) axis have been implicated in both major depressive disorder and suicide attempts (Yin et al., 2016). HPA axis dysfunction has been corroborated in living human subjects as well, with findings delineating ideators and healthy controls (Melhem et al., 2017). Interestingly, Lindqvist et al. (2008) found that increased duration of major depressive disorder has been found to result in cortisol levels being negatively correlated to “suicidal intent.” This association between SI and attempts has been found to be strongest at lower intensities of depressive symptoms (Rogers et al., 2018). Brain glucose levels have also suggested distinct neurochemical differences between those with transient SI and those who pursue suicide attempts (Sublette et al., 2013).

In spite of evidence suggesting that significant brain dysfunction is prominent in suicidal individuals across a broad range of studies and underlying or preexisting conditions (Jollant et al., 2011; Homaifar et al., 2012b), there still exists tremendous difficulties in understanding that one can predict an act seemingly based on a wide range of factors impacting current functioning, many of which are potentially beyond prediction. It is at this point that an appeal to the aforementioned scientific evidence is critical – we know that the brain, body, and external environment are intractably synchronous in their communication and reciprocal activity. Immune function is merely one example of a bodily operation that is informed and determined by neuroscience (Pariante, 2016). Therefore, if we are to establish an understanding of suicide risk and attempt quantitative prediction, we must understand the strengths, and limitations of each dimension while recognizing their mutual inclusivity.

## Limitations of Unidimensionality

Each dimension plays an integral role in STBs yet they cannot stand in isolation. Sica (2017) reported that clinical applications of observations and judgments become problematic when multidisciplinary approaches are not undertaken. Further, methodological approaches at the first phase must account for all known phases thereafter – failure of approach is often due to a lack of a forward-looking inquiry fueled by naïveté and an underestimation of the complexity of a research question. As Sica (2017) stated:

“The fault, therefore, lies in the failure to fully grasp the nature of one’s perspective and the associated limitations ecologically. The only remedy is to remain oriented toward matching the constraints (controls) in the observation and procedural adequacy regarding the clinical questions at hand.”

It is well-known that corticotropin-releasing factor (CRF) is associated with stress, fear, and anxiety, with increases in CRF resulting in hypervigilance, and other stress-related responses (Risbrough and Stein, 2006). Conversely, stress does not share a unidirectional relationship with CRF but is both a contributor to and victim of stressful situations (Ohmura and Yoshioka, 2009). In this simple example, it can be seen that a significant biological component of stress responses cannot stand alone. Similarly, an external situation beyond one’s control also cannot stand alone because the environment alters biological activity, immune response, and future behaviors.

It can also be seen that individuals react differently to stressors and environmental circumstances. The direst of situations do not imply that suicide is imminent and, instead, are seen as dependent on the degree to which one feels isolated, alone, or a burden on others (Joiner, 2005). Media coverage of tragedies can reverse the trend of feeling connected amidst tragedy, however, resulting in acute increases in suicide risk in the subsequent weeks (de Lange and Neeleman, 2004). Since Durkheim’s work in the late nineteenth century, many researchers have pointed out the flaws in viewing suicide as an outcome standing alone without mediating variables occurring within the individual – the focus was specifically on the subjective perceptions of the social world and all behaviors were attributed to that which was social rather than psychological or unique to the individual. If one were to assume that the sociological dimension was sufficient in determining a fatal downstream outcome, it would be at odds with psychological theories related to interpersonal life and the importance of simply belonging rather than the context within belongingness.

## Suicide as a Multidimensional Process

A clear, initial pathway into the discussion of a multidimensional model of suicide can be found in neurologic patients – a subset of individuals at risk of suicide who have known organic disorders of the brain. Neurologic patients are at greater risk of suicide than the general population, such as those with Alzheimer’s disease (AD; Serafini et al., 2016; Hartzell et al., 2018), epilepsy (Hara et al., 2009; Harnod et al., 2018), Parkinson’s disease (PD; Kostić et al., 2010) – particularly following subthalamic nucleus deep brain stimulation in certain studies (Voon et al., 2008; Giannini et al., 2019) – frontotemporal dementia (FTD; Zucca et al., 2018), and Huntington’s disease (Kachian et al., 2019). FTD has been found to have the highest incidence of suicide, followed by vascular dementia and Lewy body dementia (Lai et al., 2018), the latter of which is classified with PD dementia as alpha-synucleinopathies (Spillantini and Goedert, 2000). Further, behavioral and psychological symptoms of dementia (BPSD) have been found to be present in nearly 90% of AD patients, which both exacerbate symptoms, and increase the rate of disease progression (Chakraborty et al., 2019). BPSD align with well-established suicide endophenotypes, such as depression, anxiety, aggression, impulsivity, hyperactivity, disinhibition, and decision-making (Gould et al., 2017).

Neuroanatomic changes in suicide patients involve volumetric reductions, gene expression, and dysfunction in the hippocampus (Sequeira et al., 2007; Burke et al., 2018; Parizkova et al., 2018), dorsal raphe nucleus (Braun and Van Eldik, 2018; Wohlschläger et al., 2018), amygdala (Sequeira et al., 2007), and prefrontal cortex (Bani-Fatemi et al., 2018; Matsuoka et al., 2018; Mohan et al., 2018). Neurobiological findings within these regions, such as serotonergic system alterations at the receptor-subtype level, suggest that there are distinct differences between depressed suicide ideators, attempters, and those who will die by suicide (Menon and Kattimani, 2015; Sudol and Mann, 2017). While suicide is often a secondary concern for which neurologic patients are screened and treated when necessary, there are populations that may not present with depression or suicidality in its various forms. Further, the self-reports often administered to these populations assume that the individual has the capacity to recognize his or her risk – a component of perceived imminent risk that has been refuted (Van Orden et al., 2010).

### Etiopathogenesis of Suicide

Risk of suicide in neurologic populations is greater prior to significant atrophy and neurocognitive decline and these sequelae may actually serve as protective factors in later stages (Conejero et al., 2018a). If one reconsiders the aforementioned interpersonal-psychological theory, the suicide trajectory begins to resonate with a process rather than a single event that results in an unfortunate outcome. One can imagine the life of an AD patient – psychosocial stressors are present in life circumstances and one’s subjective experience of having a terminal illness cannot be predicted *a priori*. This perturbation can increase risk of suicide, which occurs concomitantly with neurobiological alterations. As the disease progresses, cognitive faculties are lost through atrophy and reduced neural connections, thereby serving as a protective factor because suicide does, in fact, require specific neurocognitive faculties such as executive functions – planning, organization, attention, and insight – as well as prospective memory.

## Extension to the Physical Sciences

It is axiomatic in the physical sciences that we reside in a universe in which the experiences around us are generally unpredictable while also being non-random. For example, one cannot predict with high specificity the events one will encounter when he or she leaves the room, nor can one similarly predict the sounds, smells, or other sensory input upon leaving the same room. These conscious and unconscious experiences may activate specific neural pathways that result in cognitive or affective alterations, such as the fear responses observed in individuals presenting with severe posttraumatic stress disorder (Diener et al., 2016; Rabellino et al., 2016). Each experience to which an individual is subjected, including interpersonal relationships (Siegel, 2012), alters the brain’s functionality. In this manner, every decision made by an individual, logically speaking, must be based on prior causes – a tendency, or predilection acquired through a previous experience or set of experiences. Unique, individual-dependent conclusions are drawn with each stimulus, but they must be processed according to the individual’s own biopsychosocial realm if they are to exist at all.

When viewed as merely an act, suicide becomes significantly more complex than the topic necessitates. When not viewed as a process, we create an inherent requirement to predict everything that surrounds an individual rather than the faculties with which an individual processes his or her surroundings. For this reason, we must focus intently on the latter. We must accept that we may be incapable of predicting the exact situation an individual may confront at any given time, but that the response to a situation will be based on the current state of neurological functioning that stems from complex interactions between every preceding moment in time. For example, the amygdala and dorsal anterior cingulate cortex (dACC) are shown to be hyperactive in fMRI studies on combat veterans (van Wingen et al., 2012; Simons et al., 2016). In these cases, impulsive and risky behaviors such as substance use are common (Gilpin et al., 2014) and point toward the unavailability of certain options that would be implied by personal agency – choosing to abstain rather than suppress craving or reward. With increased subcortical, amygdalar activity, we understand that emotion regulation (ER) techniques such as cognitive reappraisal (Heilman et al., 2010) may be unavailable due to a need for cortical engagement. In situations of hyperarousal due to posttraumatic events, the brain serves to protect itself by engaging in behaviors deemed adaptive at one point in time, even if they are maladaptive in the current moment (Harris, 2012). The dACC, both in general and in terms of its resting-state functional activity (Pannekoek et al., 2014), has been consistently implicated in the processing of emotions, and self-control (Hashimoto et al., 2015; U.S Department of Veterans Affairs, 2018).

### Application

Non-linear dynamics have been extensively studied and mathematical approaches such as algorithms have been utilized to measure effects of small perturbations in ecological time-series and forecast future changes in systems based on these previous alterations (Bar-Joseph et al., 2003; Ghadami et al., 2018). These approaches allow for prediction of unobserved points in time, yet also suggest that these same systems may exhibit non-linear kinetics (Hoffman and Galle, 2018). The question, then, is what makes study subjects such as yeast cultures different from a human being. Beyond the complex organisms human beings prove to be, humans have evolved in a non-random fashion, acquiring cognitive and affective processes through necessity rather than chance or “intelligent design.” Anthropic reasoning and self-sampling assumptions can lead one astray at this point in the process of scientific discovery. We exist because, if we did not possess the necessary features to survive in our environment, we would not exist to make the claims in the first place – it is at least possible, however, that some other life form would exist. The unique variable that is often ascribed to human beings is personal autonomy, subjecting human studies to a major flaw in empirical inquiry – the belief that humans are unpredictable and cannot be studied with the same tools utilized by the physical sciences. If we were to alleviate ourselves of this illusory concept, however, applying these properties would not seem as arbitrary or far-reaching. Instead, we may recognize that human beings are more predictable than we currently believe them to be.

As one example, the purpose of ordinary differential equations (ODEs) is to establish a framework containing the derivatives of one independent variable, which could be suicide in this particular instance. Derivatives are the values of change rate with respect to other derivatives, such as the change of mood with respect to change in living conditions or substance use, or a combination thereof. Often times, the solution to an ODE is a function rather than a number – describing this relationship – which can vary depending on availability of information pertaining to initial conditions. However, functions can serve great value in that they can define physical properties and allow for a certain degree of prediction within the universe. When applied to human behavior, mathematical approaches could serve to provide a basis for understanding complex behavior that is beyond conscious awareness, difficult to predict with judgment or isolated studies, and potentially similar across individuals.

Ordinary differential equations and its counterparts can become remarkably complex but they have been utilized to describe physical quantities and phenomena of the universe. Processes such as these are consistently utilized in fields of physics and engineering and are being revised regularly to allow for ease of use and scientific translation (Bluman and Dridi, 2012). Mathematical modeling of this kind has been applied to the medical sciences, with the goal of prediction and early detection of immune system responses to infections, and other conditions (Ilea et al., 2013; Allali et al., 2018). While this may seem different than an approach to suicide within the current psychiatric paradigm, it must be noted that immune response need not be viewed differently than neurologic responses to negative stimuli.

### Suicide as a Function of Numerous Derivatives

The act of suicide is irrefutably dependent on a broad but finite list of factors that synergistically impact one another’s roles in an individual’s susceptibility to truly engage in a suicide attempt rather than simply ideate. The Butterfly Effect, a concept originally posited by meteorologist and mathematician Edward Lorenz in 1963 (Ambika, 2015), has not been applied to psychiatric or psychological research. The concept was initially intended to predict and potentially manipulate weather conditions based on other physical properties of the universe. This is based in chaos theory serving to accurately demonstrate how small perturbations in a dynamic, non-linear system harbor the potential to result in significant deviations from a linear system over time (Ambika, 2015). This is not to say that all activities will result in major alterations to a system, but each perturbation affects every moment thereafter, with the potential for drastic changes over time that may be traced back to a seemingly inconsequential event.

**FIGURE 1:**
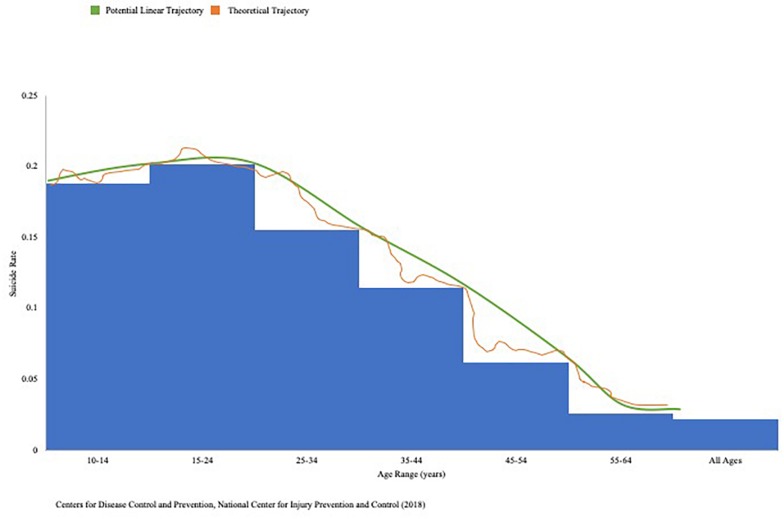
2016 suicide rates across age groups. Center for Disease Control and Prevention (CDC) WISQARS 2016 data on suicide deaths represented as ratio of total deaths attributable to top 10 causes of death in the United States, with superimposed potential linear and theoretical trajectories of suicide risk over time. This data was obtained through a unique, 1-year search via the National Center for Health Statistics, National Vital Statistics System.

Neuroscientific findings suggest behavioral, cognitive, and affective processes each play a complex, integrative, and multiplicative role in suicide. If we are to view an ideal human life as a linear system with well-being either remaining consistently optimal or increasing with time, we can more comprehensibly visualize perturbations in our chaotic universe that alter life trajectories. This may be accomplished by viewing data on suicide rates compared to the leading ten causes of death in 2016 (Figure 1). If one experiences childhood physical abuse (CPA), for example, suicide attempts are found to be significantly higher (Sachs-Ericsson et al., 2017), and often mediated by negative emotional states (Fuller-Thomson et al., 2016). This is found to also be true of the association between CPA and SI (Fuller-Thomson et al., 2012). Continuing with the graphical illustration, one instance of CPA would expectedly result in decreased well-being and increased suicide risk, dependent upon the variable of focus. Due to synergistic effects, a second instance of CPA will not cause an equal deviation from the linear system – it may cause a significantly greater increase in suicide risk. Analogously, a known protective factor such as female gender may impact this deviation, as females are less likely to die by suicide attempts than their male counterparts (Ahn et al., 2012; American Psychiatric Association, 2013).

**FIGURE 2:**
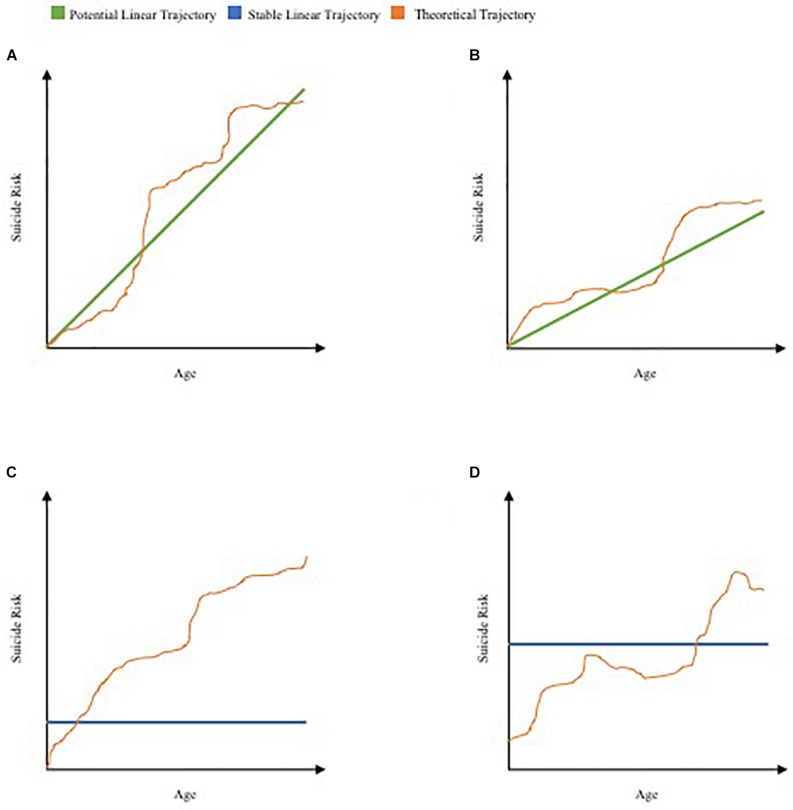
Proposed schematic of theoretical life trajectories of suicide risk. Theoretical trajectories suggested as deviations from four of infinite potential linear systems over time, analogous to graphical illustration of CDC data. **(A)** Theoretical trajectory of early suicide risk superimposed on positively correlated linear trajectory of suicide risk and age. **(B)** Theoretical trajectory of late-life increase in suicide risk superimposed on positively correlated trajectory of suicide risk and age. **(C)** Theoretical trajectory of ongoing increase in suicide risk superimposed on an ideally stable linear trajectory. **(D)** Theoretical trajectory of variable deviations in suicide risk superimposed on a second (potential) ideally stable linear trajectory.

In this fashion, the thorough and complex human studies discussed in this paper can provide necessary data to determine the degree to which certain individuals deviate from an ideal linear system following unique, often unpredictable life events (Figure 2). Studies can obtain data points such as neuroimaging, IATs, neuropsychological tests, pupillary motility (Graur and Siegle, 2013), and comprehensive metabolic panels (Bransfield, 2017) at regular intervals, substance use, medical conditions, as well as more specific, highly variable, and topically relevant data points such as instances of interpersonal interactions and passive SI, diets (Li et al., 2009; Perera et al., 2018), and daily workload (Kondo and Oh, 2010; Xiao et al., 2017). It is entirely likely that inter-individual differences will be remarkable, which is arguably one of the major causes of the lack of progress in this domain of scientific inquiry. Detailed history-gathering would be a requirement for such studies if we are to know the point at which any deviation from an “ideal” life trajectory began. Further, while suicide rates are outweighed by other age-related causes of death later in life, this does not imply within itself that suicide risk decreases – should rates of other conditions decrease, suicide rates could theoretically increase.

### Importance of Initial Conditions in Predictive Strategies

Balaguer (2004) stated that if choices appear undetermined, then they must be random or “appropriately non-random.” As previously noted, even within the field of suicide research, some scholars believe that the act itself is reliant upon such a complex algorithm of factors that accurate suicide prediction is all but impossible (Simon, 2003). If one cannot predict external reality to the extent necessary to know the experiences an individual will face when he or she carries out the day, it would, then, be impossible to predict the potential consequences of those experiences in advance. For illustration, if an individual experiences hyperreactivity to certain sounds and smells due to a past traumatic event, his or her responses to unpredictable future events could not be predicted before these events occur.

The major issue with this argument is that it regresses to a standpoint of unidimensionality. One would be hard-pressed to argue that appearance alone can provide certainty or serve as a basic premise for a syllogism. One may appear to be healthy but suffer from lung cancer while a suicidal patient may exhibit a euthymic mood only hours before completing suicide. That which is obvious to the naked eye or felt within the “gut,” which is generally a somatic response to brain activity, cannot sufficiently explain the phenomenon being observed. Further, the ability to predict external stimuli need not be discovered in order to potentially predict one’s susceptibility to certain responses following a range of possible experiences.

Due to its applications to stochastic systems, the Butterfly Effect was deemed as proof that prediction of future events is impossible due to unknown initial conditions. Even in fields such as theoretical physics and quantum mechanics, unknown initial conditions serve as an impediment to progress. For example, we currently do not have a feasible theory of quantum gravity because we are incapable of knowing what occurs at time (*t*) = 0 – classical theory is inapplicable (Modesto and Rachwał, 2017). However, human beings differ from universal properties in that human life does not begin until conception, at the absolute earliest. Beyond this point, the embryo, fetus, and eventual human is impacted by potentially infinite factors. We can state, however, that acquired mental and physical capacity for a suicide attempt is null at birth.

Extended to the human being, we need not understand the atmospheric pressure at conception to begin this type of research. Extensive history-gathering and longitudinal studies that continue to collect sufficient data could provide knowledge of ongoing conditions that may impact future suicide attempts and the degree to which they do – specifically, one’s risk at any given moment in time. Initial conditions do not necessarily impede our ability to project data points. While we cannot simply follow a linear system, we can estimate the deviation from a linear system based on a patient’s trajectory at the present moment and further determine his or her susceptibility to externalize behaviors following unpredictable life experiences. While we currently lack metadata with the necessary data points and variables, these models are viable options should we begin to embrace the external environment to which we lend so much credit from a psychological dimension.

### Computational Methods of Suicide Prediction

Determinate mathematics have often been viewed as incapable of producing reliable and complete descriptors for behaviors, even if these behaviors are deemed deterministic (Glimcher, 2003). These approaches have been studied since the early 1900s and it seemed as if axiomatic systems could not be definitively described by logical calculus or other mathematical approaches. Even when logic is complete, mathematical computations may be incomplete; if logic is incomplete, however, this in itself could serve as a logical premise (Glimcher, 2003). When applied to fields such as quantum mechanics and theoretical physics, theories exist in the form of mathematical functions that work – they may 1 day fail but they currently define observable phenomena. In this same fashion, fundamental functions of suicide risk – a result of behavioral, cognitive, and affective processes – may also be discoverable until there are better explanations, as long as they work.

It has previously been clarified that computational methods of suicide prediction, while predominantly focused on the brain, need not be criticized as a form of neuroessentialism (Datta, 2015; Gaitán and Echarte, 2016) – subjective experiences can be both appreciated and obtained as data points with a mixed-methods approach while underscoring potential eliminative materialism (Gaitán and Echarte, 2016). One of the greatest barriers to effective and clinically useful predictive measures of suicide will always be the unpredictable circumstances following examination. However, if we entertain a shift in paradigm through an appeal to the evidence, suicide risk can be viewed similar to a disease trajectory, with the major distinction being that suicide is neither degenerative nor irreversible until the final act.

## Conclusion

Suicide is not a terminal illness and this analysis does not intend to suggest that it be viewed as an inevitable result to any life trajectory. However, every neurocognitive condition is a result of the complex permutation of inputs spanning psychological, sociological, and biological dimensions, with an end result. In the case of suicide, we only view the concept as a description for this end result rather than the process leading to the behavior.

A true multidimensional, biopsychosocial model of human behavior cannot utilize one tool within the scientific toolbox and expect to construct a viable model with a strong foundation – each would commit a fallacy that attempts to support a wavering structure. If we choose to accept that which we currently understand, treating each dimension as one of many that define the universe in which we reside, we may begin to incorporate mathematical properties into the modeling of suicide and, most importantly, conceptualize suicide in a manner that appreciates its complexity as a process with a result. In doing so, suicide may be viewed by the general public as a condition devoid of stigma and worthy of attention. A medicalized, process-oriented model of suicide etiopathogenesis may be the route to altering outcomes while simultaneously ridding all fields of the belief that suicide is impulsive, unpredictable, or too complex to solve.

## Ethics Statement

In this conceptual analysis, the cited articles contain studies with human and animal work approved by the Institutional Review Boards prior to publication.

## Author Contributions

The author confirms being the sole contributor of this work and has approved it for publication.

## Conflict of Interest Statement

The author declares that the research was conducted in the absence of any commercial or financial relationships that could be construed as a potential conflict of interest.
